# Ecotoxicogenomic Approaches for Understanding Molecular Mechanisms of Environmental Chemical Toxicity Using Aquatic Invertebrate, Daphnia Model Organism

**DOI:** 10.3390/ijms160612261

**Published:** 2015-05-29

**Authors:** Hyo Jeong Kim, Preeyaporn Koedrith, Young Rok Seo

**Affiliations:** 1 Institute of Environmental Medicine for Green Chemistry, Dongguk University Biomedi Campus 32, Dongguk-ro, Ilsandong-gu, Goyang-si, Gyeonggi-do 410-820, Korea; E-Mails: hd414@naver.com (H.J.K.); pkoedrith@gmail.com (P.K.); 2 Department of Life Science, Dongguk University Biomedi Campus 32, Dongguk-ro, Ilsandong-gu, Goyang-si, Gyeonggi-do 410-820, Korea; 3 Faculty of Environment and Resource Studies, Mahidol University, 999 Phuttamonthon 4 Rd., Phuttamonthon District, Nakhon Pathom 73170, Thailand

**Keywords:** water flea, *Daphnia* spp., ecological risk assessment, ecotoxicogenomics, predictive toxicology

## Abstract

Due to the rapid advent in genomics technologies and attention to ecological risk assessment, the term “ecotoxicogenomics” has recently emerged to describe integration of omics studies (*i.e.*, transcriptomics, proteomics, metabolomics, and epigenomics) into ecotoxicological fields. Ecotoxicogenomics is defined as study of an entire set of genes or proteins expression in ecological organisms to provide insight on environmental toxicity, offering benefit in ecological risk assessment. Indeed, *Daphnia* is a model species to study aquatic environmental toxicity designated in the Organization for Economic Co-operation and Development’s toxicity test guideline and to investigate expression patterns using ecotoxicology-oriented genomics tools. Our main purpose is to demonstrate the potential utility of gene expression profiling in ecotoxicology by identifying novel biomarkers and relevant modes of toxicity in *Daphnia magna*. These approaches enable us to address adverse phenotypic outcomes linked to particular gene function(s) and mechanistic understanding of aquatic ecotoxicology as well as exploration of useful biomarkers. Furthermore, key challenges that currently face aquatic ecotoxicology (e.g., predicting toxicant responses among a broad spectrum of phytogenetic groups, predicting impact of temporal exposure on toxicant responses) necessitate the parallel use of other model organisms, both aquatic and terrestrial. By investigating gene expression profiling in an environmentally important organism, this provides viable support for the utility of ecotoxicogenomics.

## 1. Introduction

**Figure 1:**
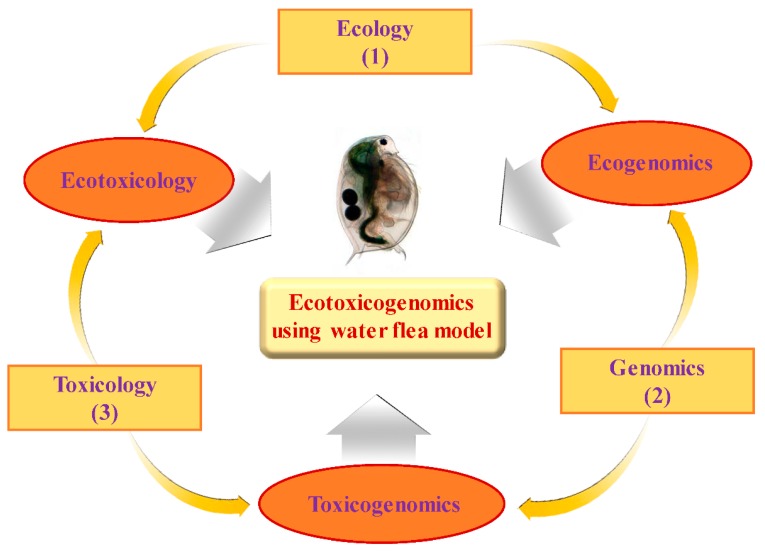
A scheme illustrating conceptual ecotoxicogenomics using *Daphnia* model. The thin arrows connect primary fields of study (in rectangles) to form interdisciplinary fields (in circles). The thick arrows indicate the tools or the knowledge that can be applied to integrated multidisplinary data sets, for instance: (1) ecological surveys; (2) genomic tools; (3) toxicity tests.

Recently, the term “ecotoxicogenomics” has been introduced [1,2,3,4,5] to describe the toxicogenomic context into ecotoxicological field (Figure 1) [6]. The toxicogenomic approach with the use of *Daphnia magna* as an aquatic invertebrate model advances our knowledge and understanding of ecotoxicity because current mechanism of chemical toxicity in invertebrates as well as potential biomarkers still need to be determined.

Basically, environmental pollutants may alter genomic expression profiles in an organism. In biomedicine, gene expression pattern is altered, directly or indirectly, owing to toxicant exposure in most cases [7]. Depending on the extent and period of the toxicant exposure, genomic alterations may be transient toxicological responses resulting in changes of “fitness” (survival and reproduction), or the “genotoxic disease syndrome” [8]. Previous studies have shown genotype-dependent effects in animals experienced to toxicants [9,10,11].

*Daphnia* is a keystone aquatic organism in the pelagic zone of most fresh water habitats (ranging from arctic and temperate lakes, lakes at high elevations, ephemeral ponds, to ponds in sand-dunes) and offers a key relationship between primary producer and higher trophic levels [12,13]. The establishment of ecology, phylogeny, toxicology, and physiology in *Daphnia* as well as its genome sequence accessibility (wfleabase.org) allow the development of genetic tools, such as genetic linkage map, [14], cDNA libraries and microarrays [6], and consequently investigate environmental impacts on gene functions in other model organisms that are difficult to be studied [15,16]. In particular, the availability of genetic linkage maps and the transferability of crossing panels across laboratories can facilitate the diagnosis of potential ecological and environmental traits via quantitative trait locus (QTL) analysis as well as the identification of heritable genotype-associated gene expression with the use of eQTL (expression QTL) approaches.

Among freshwater organisms, daphnids have relatively high sensitivity to environmental contaminants [17]. Upon exposure to environmental stressors, daphnids exhibit significant reproductive decline [18], aberrant vertical mobility and behavioral pattern, and ultimately phenoplasticity [19,20,21,22]. These abiotic and biotic stressors in common include chemical substances, synthetic hormones, acidity, salinity, calcium levels, hypoxia, radiation, bacterial pathogens, predators and parasites [16]. Among *Daphnia*’s closely related species, their multiple habitat transitions may be attributable to the extremely “eco-responsive genome” [16,23]. Among *Daphnia* closely related species complexes including *Daphnia galeatamendotae*, *Daphnia longispina*, and *Daphnia pulex*, their ecologically relevant traits are likely associated with the colonizing habitats under distinct environmental conditions [24,25,26]. The multiple lineages independently colonized and adapted to these freshwater habitats are distinguished in terms of extent of reproductive isolation and intraspecific genetic subdivision among populations [27,28].

**Figure 2:**
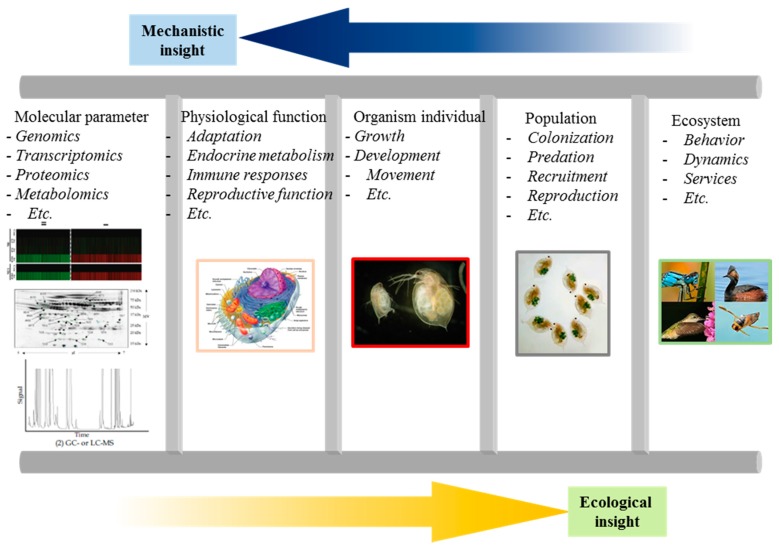
A scheme representing multilevel-framework for ecotoxicogenomic studies at multiple levels ranging from molecular, physiological, organismal, and population in ecosystem.

In the field of molecular biology at post-genomic era, the availability of DNA sequence data combined with advent in genomic tools and technologies will promote the direct interrogation of gene expression at multiple levels in organisms experienced to various environmental stressors. Given that the genome sequencing tool has the potential to identify an increased number of ecologically relevant species in both vertebrates and invertebrates, this holds great promise to address the phenotypic and genotypic linkage based on fitness using a “bottom-up” approach from molecular to ecosystem level (Figure 2) [2].

Genomic tools can facilitate ecology and evolutionary biology studies, allowing advance fundamental information and addressing the future issues related to chemical effects on environmental and human health. Purpose of this framework include: identification of ecological performance-regulated gene loci; functional analysis of ecological performance-related traits; evaluating individual, population, community, and ecosystem responses to the environment; examining the degree and significance of genetic variation among ecological performance-related traits [2].

Of ecological significance, omics technologies including microarrays enable rapid quantification of molecular variation among populations at multiple gene loci [2]. In the UK, the Natural Environment Research Council (NERC) Environmental Genomics Programme has been established in order to: examine the consequence of genetic variation on ecological performance; determine the degree of spatial and temporal variation at regulatory *versus* structural loci; investigate numbers of loci and their genomic distribution; and assess community structure and the influence of environmental alteration. At environmental contaminated sites, the omic tools are also useful for investigating important issues as follows: what are inducible genes and their functions; is there variation in gene expression in response to environmental change; is the variation adaptive; what are the consequences of the genetic variation-mediated molecular transformations at ecosystem-, community-, and population-level.

The omics-based technologies can facilitate comprehensive investigation to better understanding and improve the knowledge on how environmental stressors, such as heavy metals and persistent organic pollutants, cause toxicity in ecologically relevant organisms and adverse effects in ecosystem [29]. In this review, we mainly discuss three aspects: the commonly used omic technologies, including genomic (or mRNA-transcriptomic), proteomic, metabolomic analyses, and more recently emerged epigenetic technology; collective technologies (*i.e.*, cDNA microarray, high-density oligonucleotide arrays, suppression subtractive hybridization PCR or high-throughput pyrosequencing, two-dimensional gel electrophoresis, fluorescence difference gel electrophoresis, ProteinChip, surface-enhanced laser desorption ionization (SELDI) mass spectrometry, and nuclear magnetic resonance) in conjunction with statistical testing or multivariate analysis; as well as development of bioinformatics tools and their application to aquatic ecotoxicology studies.

### 1.1. Practice of Ecotoxicogenomics in Genomic or Transcriptomic Responses

The DNA microarrays applied to ecotoxicology have ranged from nylon membranes that are custom spotted with a couple dozen selected cDNAs [30,31,32] to commercially available, high-density arrays consisting of thousands of oligonucleotides synthesized directly onto a solid support such as a glass slide [33,34,35]. To date, the availability of commercial, high-density oligonucleotide microarrays has been largely restricted to prominent model organisms for which considerable sequence information is available, such as zebrafish (*Danio rerio*), frog (*Xenopus larvis*), and fathead minnow (*Pimphales promelas*). Although the genomes of other species have not been completely sequenced, DNA microarrays have been developed and applied for studying a variety of additional model organisms for ecotoxicity testing. Coordinated efforts to share sequence information and other resources, such as the *Daphnia* Genomics Consortium (http://www.daphnia.cgh.indianna.edu/) and the Consortium for Genomic Research on All Salmon (http://www.web.uvic.ac/grasp/), have greatly assisted the development of genomic tools for other model organisms. Up-to-date, high density customized microarrays of cDNA- or oligonucleotide-based chips containing thousands of targets have been dominantly developed for global analysis of transcripts associated with exposure to chemical or environmental stressors in ecotoxicology studies.

The emergence of transcriptomics as a tool for ecosystem characterization is perhaps best shown by the fact that transcriptomics has been applied to a diverse group of species and stressors, representative of those traditionally examined in context of ecological risk assessment [29]. As a common test organism, the aquatic invertebrate, *D. magna* has also been used in a number of ecotoxicology-oriented transcriptomics studies [36,37,38,39]. To date, *Daphnia* and fish, particularly zebrafish and fathead minnow, appear to be the most common model organisms for ecotoxicogenomics studies employing DNA microarrays. As DNA sequencing technology becomes increasingly rapid and cost effective, the available sequence information for diverse taxa continues to grow, making it increasingly feasible to apply transcriptomic approaches to nearly any species of concern [40].

For example, the first version cDNA microarray of *D. magna* based on Suppression Subtractive Hybridisation PCR (SSH-PCR) 855 life stage-specific cDNAs has been successfully applied for elucidating mode of toxicity of propiconazole pesticide [41]. After 4 days of exposure to 1 μg/mL propiconazole, vitellogenin gene was repressed, suggesting that oocyte maturation was affected. The vitellogenin mRNA might be considered as an early warning biomarker of chronic reproductive effects in aquatic invertebrates. Metal toxicity study was performed using a customized *Daphnia magna* cDNA microarray toward sublethal exposure to cadmium, copper, and zinc [37]. This finding revealed that distinct expression patterns toward each metal, and plausible exposure biomarkers, including putative metallothioneins and ferritin mRNA with a functional IRE were identified. Furthermore, using systems biology approach, transcriptomic patterns and phenotypic features were analyzed in *D. magna* model following exposure to ibuprofen, a nonsteroidal anti-inflammatory drug [36]. Result indicated that ibuprofen would affect reproductive capability at molecular-, organism-, and population-level in daphnids. Microarray data demonstrated ibuprofen-mediated early disturbance in crustacean eicosanoid metabolism via signal transduction, resulting in aberrant juvenile hormone metabolism and oogenesis. Recently, microarray analysis in combination with reproduction testing assay was conducted in *D. magna* system in order to determine bisphenol-A(BPA) toxicity since BPA has detrimental health impacts, particularly in reproduction, development, and organismal behavior [42]. Microarray results revealed significant change in expression levels of candidate genes homologous to animal nucleotide sequences, including cuticular protein, vitellogenin, protease, and ribosomal proteins. These genes reportedly discovered in other animal models might be recognized as novel biomarkers indicative of BPA exposure [42,43]. The application of genomic techniques into environmental toxicology holds great promise to identify exposure biomarkers and clarify the mode of toxicity of newly synthesized chemicals [44]. Using 15k oligonucleotide microarray, *D. magna* was also used as an aquatic model to investigate nanotoxicity, specific biomarkers, and effects of coating agents; for instance, citrate-coated and polyvinylpyrrolidone (PVP)-coated silver nanoparticles (AgNPs) in comparison to bulk silver nitrate (AgNO_3_) [44]. The microarray data showed distinct expression patterns toward AgNPs and AgNO_3_, indicating distinct modes of toxicity. AgNPs affected to biological processes, especially protein metabolism and signal transduction whereas AgNO_3_ suppressed sensory developmental processes. Only PVP-coated AgNPs could upregulate metal-and DNA repair-related genes. PVP-coated AgNPs-specific biomarkers, including metallothionein (MT) and DNA damage repair (REV1) gene might be useful for the environmental detection.

Regardless of the stressor(s) examined, the most prominent use of transcriptomics has improved the understanding of the mechanisms of action through which various stressors elicit or modulate adverse effects [29]. Mechanistic ecotoxicogenomic studies rely heavily on genome annotation to identify the specific transcripts modulated by exposure to a particular stressor and gain an understanding of the biological functions and pathways represented by the differentially expressed genes. Qualitative and quantitative analysis of “enriched” gene ontology (GO) terms tends to be one of the central analytical approaches used in mechanistic studies. Application of automated pathway analysis tools has been somewhat limited by the lack of standardized pathways for ecological model species (*i.e.*, non-human, non-rodent). However, pathway-oriented analyses can still be conducted by hand [45,46] or by using bioinformatics approaches to identify human or rodent homologs and applying pathway tools developed for those species [40,47]. Ultimately most mechanistic transcriptomic studies have had to rely heavily on information available in the extant literature to make sense of the numerous, and often disparate, responses that are observed following exposure to a stressor.

Transcriptomic studies have already produced a greater appreciation of the concept that exposure to stressors, even those traditionally thought to act through specific pathways, impacts on many fundamental cellular processes such as energy metabolism, protein metabolism, cell cycle, cytoskeletal organization, immune/inflammatory processes, and extracellular matrix development [34,48,49]. Microarray studies suggest that extensive crosstalk among pathways is perhaps the rule, more than the exception. Given the global nature of microarray analysis, one of the key challenges of mechanistic studies is differentiation of general stress responses from responses that are specific to a given mode or mechanism of action [50,51,52]. Furthermore, transcriptomic research appears to be enhancing appreciation for adaptive or compensatory responses to stressors as well [45,53,54]. The ability to differentiate adaptive responses from adverse ones is critical if transcriptomic responses are to be used as a basis for predictive ecological risk assessment.

Toxicogenomics researchers have clearly recognized the need for phenotypic anchoring to link changes at the molecular level to outcomes at higher levels of organization. The majority of mechanistically oriented transcriptomic studies in ecotoxicology have included a variety of apical endpoints. For example, transcriptomic results for *Daphnia magna* were anchored to embryo abnormalities and carapax length in one study [55] and population growth rates in another [39]. An ecotoxicogenomic assessment of *D. magna* with the use of expressed sequence tags (ESTs) and the database has been conducted [56]. Based on this sequence information, an oligonucleotide-based DNA microarray has been developed in order to determine the acute toxicogenomic profiling of *D. magna* in response to various types of chemical stressors, including copper sulfate (CuSO_4_), hydrogen peroxide (H_2_O_2_), pentachlorophenol (PCP), and β-naphthoflavone (βNF) as testing substances exerting distinct toxicities. The result showed that neonatal daphnids exposed to these compounds have distinct transcriptional changes toward each chemical. However, it is necessary to relate molecular mechanisms to toxicological outcomes when mechanistic ecotoxicogenomic studies in aspect of “proof-of-concept” are conducted.

**Figure 3:**
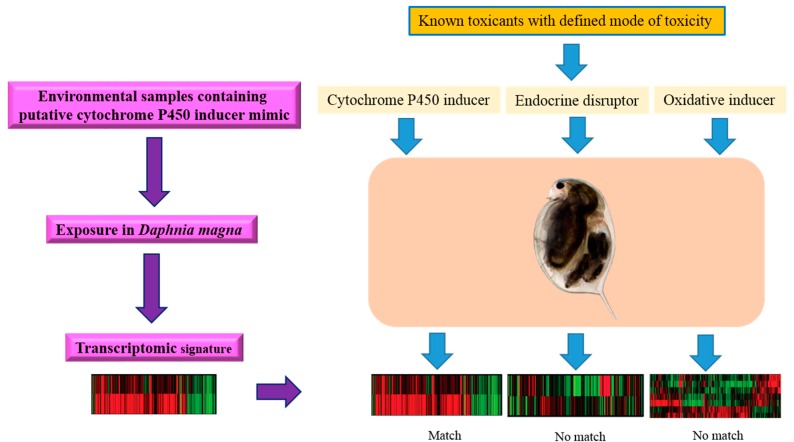
A scheme illustrating the utility of gene expression signatures to explore the possible mode of toxicity of an unknown environmental toxicant in an aquatic model organism (*Daphnia* spp.).

While ecotoxicogenomic studies have tended to focus on mechanisms, mechanistic research has not been conducted to the exclusion of fingerprinting approaches and biomarker discovery (Figure 3) [2]. Many in the field have used their transcriptomic data to both explore mechanisms and attempt to identify transcriptional fingerprints or biomarkers potentially indicative of specific types of exposure or effects. For example, Poynton *et al.* [37] identified distinct expression profiles in *Daphnia magna* for three different metals, found support for known mechanisms of metal toxicity, and postulated inhibition of chitinase activity by zinc as a novel mode of action [37].

Alternatively, techniques for high-throughput transcription analysis that are not relied on a priori information of DNA sequence database include suppression subtractive hybridization PCR and high-throughput pyrosequencing [57]. Nevertheless, the data interpretation somewhat depends on the knowledge of genome like that in microarray techniques. Several setbacks of the microarray techniques may thus be employed to the aforementioned techniques.

This notwithstanding, researchers can predict that, except for genome model species, only a 5%–10% of the genome can be related to known genes and thus to defined functions. New functional data, novel developments in bioinformatics, and completion of sequencing projects will be needed to conciliate the spectrum of species and taxa required for an in depth analysis of environmental impacts, and the limitations of transcriptomic and functional analyses.

Even in laboratory model organisms, much effort is still needed for gene functional analysis. In the genetic organism models (e.g., *Saccharomyces cerevisiae*, *Caenorhabditis elegans*, *Drosophila*, and *Mus musculus*), gene functions can be analyzed on the phenotypic characterization of mutants, transgenic organisms using common techniques (*i.e.*, knockdown or gene silencing, knockouts, and the reverse genetics method [58]. Some of these conventional genetics methods become available in *D. magna* [59,60], ecotoxicogenomics-based approaches would be complementary with the validation of results obtained by those techniques. Ecotoxicogenomics-oriented research in such small organisms like the water flea may help to overcome the difficulties in the higher levels of ecological organisms in terms of studying the whole organism.

### 1.2. Practice of Ecotoxicogenomics in Proteomic Response

To date, proteomics has been the second most widely used of the omics approaches applied to ecosystem assessment [40]. Proteomic studies account for around 10% to 15% of published ecotoxicology-oriented “omics” research. Most of the proteomics studies have focused on fish and mussels.

In contrast to transcriptomics-based ecotoxicology studies, which, to date, tend toward mechanistic investigation, proteomics-based ecotoxicology has skewed heavily toward identification of fingerprints, most often referred to as protein expression signatures (PES). There are two dominant practical approaches to PES generation. ProteinChip technology along with SELDI mass spectrometry, also known generally as retentate chromatography-mass spectrometry (RC-MS) [61], is the most common analytical technique used for PES identification. The second most prominent is two-dimensional gel electrophoresis and matrix-assisted laser deionization (MALDI)-based mass spectrometry has also been employed.

Laboratory-based proof-of-principle type studies aimed at PES identification have shown some promise. Early on, this primarily consisted of work that employed two-dimensional gel electrophoresis (2-DE) and showed that exposure to different chemical treatments resulted in distinct spotting patterns on the gels [62,63,64]. While these studies provided basic support for the hypothesis that exposure to a given stressor would yield a unique profile of protein expression, limitations associated with the technical reproducibility of 2-DE were a significant barrier to wide applications of the technique for environmental, or even laboratory, diagnostic applications [65]. The second generation of proteomics studies aimed at identification of PES has made greater use of more sensitive and reproducible technologies, for example, using SELDI time-of-flight (TOF) mass spectrometry (MS) (SELD-TOF MS), fluorescence difference electrophoresis (DIGE) and MS, SELD-TOF MS with a weak cation exchange ProteinChip, and either SELDI-TOF MS or MALDI-TOF MS in conjunction with ProteinChip arrays. At present, the bulk of published evidence suggests that, at least under controlled laboratory conditions, exposures to various types of stressors can be discriminated based on proteomic profiles in a variety of sample types.

While laboratory-based discrimination of stressors may have utility for prospective assessments, diagnostic assessment requires methods that can be applied to samples collected from less controlled field conditions [40]. A number of studies have investigated the applicability of proteomic approaches to field-based assessments, for example, using SELDI-TOF MS and a ProteinChip with a strong anionic exchange surface. To date, the evidence collected broadly supports the notion that PES can be used to discriminate among sites with differing stressor profiles.

The current body of work establishes an effective proof of principle, that is, stressors elicit changes in the proteome that can be detected and used as a basis for class discrimination [40]. It remains to be seen whether the discriminatory capabilities of proteome fingerprinting, demonstrated in the laboratory and field, will ultimately be useful for risk assessment. To date, we are not aware of any studies that have examined whether specific PES or proteomics-based class separation models identified through statistical testing or multivariate analysis, such as principal components analysis (PCA), partial least squares discriminant analysis (PLS-DA), and neural networks can be consistently reproduced and successfully applied in multiple independent laboratory or field studies. Few proteomics-based ecotoxicology studies have examined the degree to which PES vary as a function of either time or stressor concentration/severity. Additionally, the influence of confounding environmental variables such as temperature, habitat quality, diet, predator/prey, or host/parasite relationships have not been examined.

Researchers in the field of environmental proteomics are aware of the ongoing need to generate greater confidence in the accuracy, reproducibility, sensitivity, and robustness of the methods before they are integrated into risk assessments [66]. As such, the ongoing challenge relative to application of PES in ecological risk assessment is not only identifying proteomics-based classification models that can discriminate among various groups of samples, but evaluating and establishing the specificity and robustness of those models across widely varying experimental or environmental conditions are also of concern.

For example, using the two-dimensional gel electrophoresis (2-DE) technology impacts of toxic heavy metals such as As(III), As(V) and Cd and their binary mixtures on proteomic profiling in *D. magna* were assessed in order to explore novel protein biomarkers [67]. The result demonstrated that 117 totally altered proteins might be recognized as plausible protein biomarkers in *D. magna* for sensing heavy metals in the aquatic ecosystem. It also suggested that binary mixtures of heavy metals in *D. magna* present somehow molecular interactive complexity, rather than simple sum of the proteomic profiles of the individual metals. Using a redox-focused proteomic approach by labeling protein carbonyls with fluorescein-5-thiosemicarbazide (FTC) prior to 2-DE, proteomic profiling and molecular biomarkers were successfully evaluated; meanwhile whole organism-based toxicity and biochemical testing were analyzed in *D. magna* exposed to citrate-coated silver nanoparticles (AgNPs) compared to bulk silver nitrate (AgNO_3_) [68,69]. Levels of vitellogenins were increased upon exposure to both compounds, suggesting their functionally overlapped general stress response. Hemoglobin levels were increased toward AgNP exposure whereas 14-3-3 protein (a regulatory protein) carbonylation levels were decreased upon AgNO_3_ exposure, indicating that both silver compounds has distinct impact on biological pathways possibly resulting in differential interactions with either natural or xenobiotic substances in the aquatic environment. 

### 1.3. Practice of Ecotoxicogenomics in Metabolomic Response

Among the “omics” approaches, metabolomics has been the least widely applied to ecotoxicology [40]. Whereas transcriptomics and proteomics attempt to examine the entire complement of transcripts or proteins, respectively in a sample, metabolomics is concerned with the complement of small molecule metabolites found in biological samples [40]. Metabolomics studies account for approximately 5% of published ecotoxicogenomic studies. The majority of these have employed nuclear magnetic resonance (NMR) spectroscopy as the primary analytical technology. However, mass spectrometry (MS)-based environmental metabolomics studies are beginning to appear in the literature as well [70,71,72].

Similar to proteomics, the majority of ecometabolomics studies have focused on demonstrating that distinct metabolic profiles are observed for organisms exposed to different types of chemical stressors either in the laboratory or in the field [40]. Multivariate pattern-recognition analyses are typically used to assess complex differences between spectra. Thus, studies with a variety of species and stressors have demonstrated the ability to discriminate among groups using metabolite profiles, and to identify specific metabolites that may serve as biomarkers. However, to date, we are not aware of any concerted efforts to develop libraries of “metabolic fingerprints” for use in identifying or classifying various types of exposures or effects.

When specific metabolites that were altered by a chemical exposure or differed among populations exposed from different (contaminated) environments can be identified, metabolomics studies also have the potential to yield mechanistic information [40]. Cautions have been raised that ad hoc attempts to infer mechanisms, based on metabolomic data in isolation, are not likely to be successful [73].

For example, an effective approach of high throughput, ultrahigh resolution mass spectrometry based metabolomics namely “direct infusion Fourier transform ion cyclotron resonance mass spectrometry (DI FT-ICR MS)” has been established as an exceptional tool in *D. magna* [74]. Copper was used as a testing chemical to validate this technique with an OECD 24 h acute toxicity testing in both univariate and multivariate models in order to screen and prioritize chemicals within tiered risk assessment. Later work with use of FT-ICR MS based metabolomic approach was successfully validated with *D. magna* toxicity testing to evaluate the acute metabolomic effects of chemicals and their mode of toxicity [75]. Test compounds with distinct toxicity modes including cadmium (oxidative stress inducer) [76], fenvalerate (sodium channel activator) [77], 2,4-dinitrophenol (DNP) [78], and propranolol (nonselective β-blocker) [79] were employed to evaluate whole-body metabolome relative to hemolymph metabolome with use of supervised multivariate modeling. The finding indicated that metabolomic patterns derived from whole-daphnids have discriminatory accountability to MOA of chemicals rather than hemolymphs as well as early metabolomic responses enable reflect discriminatory acute toxicities of chemicals. Furthermore, the integration of hemolymph metabolomics namely “FT ICR MS and NMR spectroscopy” and whole-daphnid transcriptomics namely “*D. magna* 44k oligonucleotide microarray” based method in conjugation with use of KEGG pathway database and gene ontology offers holistic insight on how cadmium at sublethal concentrations for 24 h interrupt nutrient uptake and metabolism. This thus led to impaired energy production, resulting in chronic toxicity [80].

Recently, ^1^H NMR-based metabolomics has been applied as viable platform for investigating metabolomic profiling and mode of toxicity of *D. magna* upon exposure to toxic metals including arsenic, copper, and lithium at sublethal concentrations for 48 h [81]. Metabolomic responses under all treatments were statistically compared using principal component analysis (PCA), and differentially expressed metabolites were quantitatively identified. Metabolomic data indicated that lithium exposure significantly exhibits an analogous mode of toxicity to copper as evident by disrupted energy reservoir and regulation, while arsenic exposure has a metabolic shift with non-significant changes.

### 1.4. Practice of Ecotoxicogenomics in Epigenetic Response

Epigenetic effects can be defined as inheritable changes in phenotypes, by either mitotically or meiotically, without changes in DNA sequence [82]. Gene expression changes can be mediated via well-studied processes including DNA methylation, histone modifications, and RNA interference as well as less well-studied epigenetic processes (*i.e.*, histone variation, nucleosome phasing, higher-order chromatin structure organization, and nuclear localization [83,84].

DNA methylation, which is occurred by either *de novo* or maintenance DNA methyltransferases, is related with transcriptional regulation, chromosome inactivation, and transposable element regulation [85]. Even though DNA methylation is present in various eukaryotic organisms, the degree of methylation and the chromatin structure organization are dependent on species and developmental stages [83]. DNA methylation interacts with other epigenetic processes including histone modifications at amino- or carboxyl-termini, thereby affecting chromosome coiling and accessibility to transcriptional machinery and ultimately resulting in gene expression changes [86,87]. Additionally, DNA methylation and histone modifications can interact with the RNA interference (RNAi) system that is involved in the generation of small noncoding RNA molecules (ncRNA) [88]. The nc RNAs, such as microRNA (miRNA) and short interfering RNA (siRNA) can form RNA-induced silencing complexes (RISC) that recruit DNA methyltransferases and histone modifying enzymes [89].

Epigenetic markers are regulated by environmental conditions (*i.e.*, nutrients, chemical stressors, hypoxia, and developmental stages) [90]. For example, histone methylation status can be modified by hypoxia via interruption of Jumonji protein (JMJD2) activity in aquatic system [91]. Indeed, the term “epigenetics” can be simply described to any environmentally modified process of DNA, such as DNA methylation or histone modification regardless to maternal inheritability. For example, previous studies revealed that transgenerational incidence is unlikely related to methylation status in either *Daphnia* [92] or *Fundulus* [93].

These epigenetic mechanisms in normal *Daphnia* development and their adaptations remain to be elucidated. Vandegehuchte *et al.* [94] have firstly discovered that *D. magna* is capable of methylating DNA as well as genes homologous to major vertebrate DNA methyltransferases (Dnmt1, Dnmt2, and Dnmt3A) with their activities confirmed [94,95]. Using ultra-performance liquid chromatography (UPLC) and microarrays, DNA methylation and transcriptome profiling were respectively analyzed in *D. magna* exposed to several chemicals [96]. This finding showed that global or localized DNA methylation levels could be altered by 5-azacytidine, vinclozolin, genistein, and zinc but not by 5aza-2′-deoxycytidine, biochanin A, and cadmium [94,96]. The transgenerational impacts of methylation status were also determined [96].

With available accessibility of complete genome for *D. pulex* and *D. magna*, the advanced techniques, such as bisulfite sequencing, methylated DNA immunoprecipitation (meDip), or DNA methylation sensitive restriction enzyme digests preferably enable measurement of methylation status of particular genes, offering biologically meaningful information. Using these methods, *D. magna’s* certain genes related to growth and reproduction were definitely determined since body length, brood size and sex determination as well as helmet and neck-teeth are affected upon exposure to toxicant [92,94,96,97,98,99,100].

Regarding to *Daphnia* epigenetics in DNA methylation process, CpG methylation is occurred at relatively low level but it is sensitive to developmental stage, as evident by the 2-fold increase in percentage of CpG dinucleotides in adults at 32-day age compared to that at 7-day age [101]. In epigenetic aspects in other core processes (*i.e.*, histone modification or noncoding RNA), or the impact of these epigenetic mechanisms on either normal development or the well-known predator-induced epigenetic polyphenisms, this information is still lacking. For example, previous investigation reported that both histone H3 and H4 modifications were occurred in embryonic cells. Interestingly, histone H3 dimethylated at lysine 4 (H3K4me2) was non-uniformly present in a cell-cycle-specific manner in *D. magna* gastrula cells but was absent in oocytes.

## 2. Suggestion of Promising Biomarkers of Environmental Toxicity or Exposure

**Figure 4:**
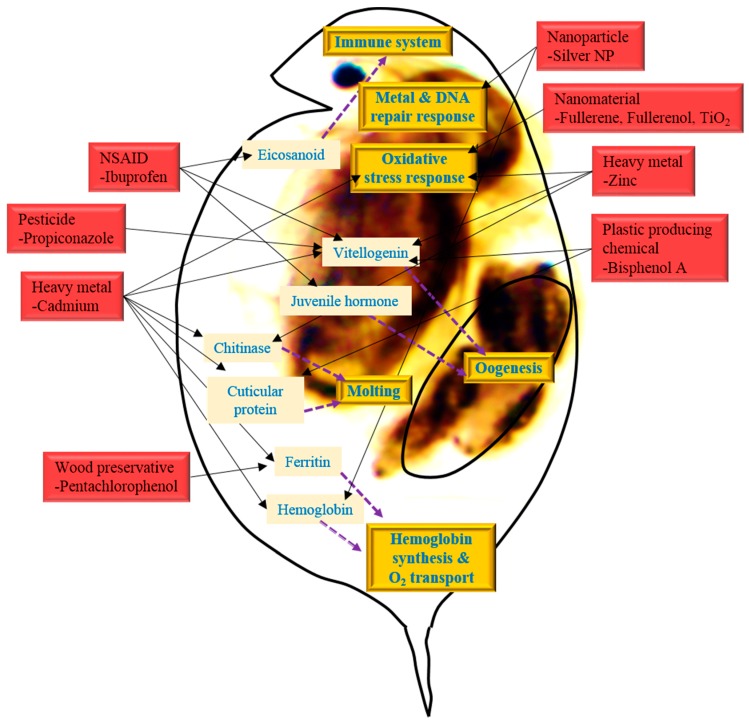
A scheme showing overview of biomarkers and biological interactions in *Daphnia* in response to stressors (as indicated 3-D red square). These candidate genes might be considered as potential biomarkers (as indicated in pink square), and their products are involved in important biological processes (as indicated in 3-D orange square) via interference with certain biomolecules (as indicated in 2-D pink square).

The toxicogenomic studies using *Daphnia* have potential advantages not only to identifying interlinked crosstalks and biological processes in response to environmental toxicants, but also to suggest promising biomarkers that are indicative of certain types of environmental stressor’s effect or exposure. Indeed, this recognizes the need for phenotypic anchoring to link changes at the molecular level to outcomes at higher levels of organization. The majority of mechanistically oriented transcriptomic studies in ecotoxicology have included a variety of apical endpoints. In various studies about toxicity using *Daphnia* as an aquatic test model to metals, endocrine disruptors, drugs and so on, data enables us to understanding the symptoms induced by those toxicants. Although the detailed pathways and mechanisms of the toxic effects have not been clearly elucidated, *Daphnia*-customized microarray data, in particular, reveals a number of biomolecules including genes involved in ion transport and chelating (ferritin, putative metallothionein), metamorphosis (vitellogenin and chitinase), invertebrate immune system (eicosanoid), glycolytic and proteolytic process (amylase, cellulose, esterase, and serine protease), cellular anti-oxidative defense (glutathione-*S*-transferase, catalase, and peroxiredoxin), and stress response (heat shock proteins) that have been predominantly recognized as suggestive biomarkers in response to environmental stressor’s exposure or effect (Figure 4) [37,38,39,102,103].

Heckmann *et al.* [104] revealed the profiles of critical genes in daphnia caused by ibuprofen exposure using microarray and temporal real-time quantitative PCR. In acute ibuprofen exposure, several genes, such as Lip (triacylglycerollipase), Ltb4dh (leukotriene B4 12-hydroxydehydrogenase), FABP3 (fatty acid binding protein 3), DmagVTG1 (vitellogenin 1), dmHb (hemoglobin), JHE (juvenile hormone esterase), VMO1 (vitelline outer layer membrane protein 1), and Cht (chitinase) are up- and down-regulated, inducing or suppressing the metabolisms and cellular processes. Lip and Ltb4dh are linked to eicosanoid metabolism and play a vital role in the invertebrate immune system, and FABP3 induce Peroxisome Proliferator Activated Receptor (PPAR) signaling pathway in the endocrine system. A temporal expressed gene, Cht is a key enzyme to molting fluid enzyme secreted during apolysis [105]. JHE, DmagVTG1 and VMO1 is related to oogenesis of daphnia, and separately expressed at different times in the developing oocyte [104].

Cadmium exposure microarray data are also analogous to ibuprofen exposure data, inducing glycolytic, proteolytic, homeostatistic, heat shock protein genes, and oxidative stress responses. Up-regulated Cht gene encoding chitinase and CYP450 gene encoding Cytochrome P450 monooxygenase, down-regulated LDLa encoding low-density lipoprotein receptor domain class A, and DD5 gene encoding a cuticular protein are involved in endocrine system of molting. In addition, up-regulated ferritin 1-like protein A and hemoglobin 2 (BJ9311841) and down-regulated hemoglobin1, 2 (BJ932805), 3 are linked to iron storage and iron absorption. Up-regulated ferritin genes in *D. magna* have previously been associated with cadmium exposure [37,102]. The general stress response gene HSP70 is also up-regulated [39].

The concentrations and time periods have an effect on different gene expression profiles. Soetaert *et al.* [102] stated that alteration of gene expression resulted from high concentration of cadmium. According to performed microarray data, 266 genes changed their expression after cadmium exposure. The genes are related to several cell processes including digestion, oxygen transport, vitellogenin, cuticular metabolism, immune response, acid-base balance, visual-sensory preption and signal transduction. The up-regulated genes, cellulase (DW724578), alpha-amylase (DW985556) and alpha-esterase (DW724473), induce digestive processes of carbohydrates and lipids. Hemoglobin (dhb1) (DW724693) and di-domain hemoglobin precursor were down-regulated, resulting in transport of less O_2_. Furthermore four genes encoding vitellogenin, two of which are well documented in *Daphnia magna* [vitellogenin1 (DY037262) and vitellogenin2 (DY037256)], were also down-regulated. Seven genes related to cuticula metabolism showed altered expression [102].

In the case of cadmium and copper exposure, *D. magna* ferritin gene (AJ292556) was up-regulated. Putative metallothionein1 (MT1) cDNAs, DV437826 and DV437799, were up-regulated by cadmium exposure and copper and cadmium co-exposure [37]. MT proteins are involved in detoxification of heavy metals and have been used as biomarkers of metal exposure for many years [106].

For CuSO_4_ or H_2_O_2_ exposure, the genes had similar expression pattern. Both chemicals are known to induce oxidative stress [107,108,109,110]. CuSO_4_ and H_2_O_2_ induced gene expression of glutathione-*S*-transferase (GST), serine proteinase inhibitor (Serpins), and alcohol dehydrogenase (ADH). CuSO_4_ also up-regulate lysosomal thiol reductase and expressed sequence tag, the flamingo homolog was up-regulated by H_2_O_2_ exposure. Pentachlorophenol (PCP) exposure induced ferritin-encoding gene, and beta-naphthoflavone induced gene encoding cathepsin L-like protease [38].

GST is also the biomarker of exposures to nanoparticle, fullerene. Catalase (CAT) is also the biomarker of nanoparticles. These two proteins involved in the detoxification processes during oxidative stress, are commonly used biomarkers in toxicity tests and indicate the early alteration of response to pollutants. The GST and CAT levels were induced in fullerene nC_60_, fullerenol C_60_(OH)_24_, and titanium dioxide (TiO_2_) exposure by the daphnids. Fullerene related to functionalization of nanoparticles and TiO_2_ is the core structure of the nanoparticles. Both functionalization and core structure affect GSH and CAT expression [103,111,112].

In a study focusing on Zn exposure, large numbers of genes were up- and down-regulated. Transcription- and translation-related genes and all vitellogenin-related genes were down-regulated while oxidative stress responsive genes (such as glutathione-*S*-transferase and peroxiredoxin) and other different types of metabolism-related genes were up-regulated, in comparison to the control [92]. Ribosomal proteins were both up- and down-regulated [92].

In a study of BPA toxicity using microarray techniques and Blast2GO, Jeong *et al.* [42] elucidated the molecular mechanisms by altered genomic responses, the molecular functions, and biological processes. They selected several candidate biomarkers which were related to BPA exposure. The reproductive activity of *D. magna* has been related to the function of molting and chitinase activity [105]. Genes related to chitin activity, chitin deacetylase 2 isoform A (NP_001096047), chitin binding domain-containing protein (ABU80624), chitin binding protein 4 (BAI44118), and encoding cuticle protein (cuticle protein 1b) were up-regulated. Serine protease encoding gene, serine protease 13 (ABZ04021), serine protease H82 (EFA11957), and serine proteinase stubbles (EFN83787, EGI65883), were also up-regulated. Various proteolytic enzymes, such as serine protease can be affected molting cycle of *Daphnia* causing degradation of exoskeleton [102,113]. When vitellogenin is fused with superoxide dismutase, it is down-regulated and influences embryo development and reproduction of daphnia by providing nourishment for growing embryos [114].

## 3. Adverse Outcomes from Environmental Stressor Exposure

Various toxicants such as endocrine disrupting chemicals, heavy metals, pharmaceutical drugs, and organic compounds that are widely distributed in environment can perturb the ecologically relevant organisms. These toxicants disturb ecosystems by influencing organisms at the bottom of the ecological pyramid. When the organisms are affected, the entire ecological pyramid can collapse. Therefore the base of ecosystem is crucial for the entire web of living things, including humans. Studies on the toxic effect using the daphnia systems have been carried out recently. *Daphnia* is a model species to study aquatic environmental toxicity designated in OECD toxicity test guideline [115,116].

Endocrine disrupting chemicals (EDCs) interact with various receptors, such as the estrogen receptor (ER), androgen receptor and aryl-hydrocarbon receptor [117,118]. EDCs have an effect on reproduction by reducing the proportion of juveniles and by producing the male daphnia [119,120,121]. Heavy metal contaminants usually have negative effects on the development of population growth rate, longevity, and reproduction [6]. Organic compounds and drugs also have detrimental effects on reproduction and development impacting fecundity, abnormality of embryogenesis, and molting [104,122]. Table 1 summarizes studies of the effects of toxicants (*i.e.*, EDCs, heavy metals, pharmaceutical drugs, organic compounds, and nanoparticles) on daphnia system.

**Table 1:** Toxicological studies using daphnia system in response to potential environmental stressors including endocrine disrupting chemicals (EDCs), heavy metals, organic compounds, pharmaceutical drugs, and nanoparticles.

Chemicals	Species	Chemical Exposure	Effect	Reference
EDCs	*Daphnia magna*	Nonylphenol at 0.024 mg/L for 48 h	Abnormal proportion of juveniles	[119]
	*Daphnia magna*	Juvenile hormone at 330 ng/L for 21 days	Production of male offspring	[121]
	*Ceriodaphnia dubia*	Styrene at 0.04–1.7 μg/L for 7 days	Mortality and reduced fertility	[120]
	*Daphnia magna*	Bisphenol A at 6.67 and 10 mg/L for 21 days	Reduced offspring production	[42]
Heavy metals	*Daphnia magna*	Cadmium at 6, 20, and 37 μg/L for 24 h	Reduced survival and somatic growth	[39]
	*Daphnia magna*	Cadmium chloride at 71 μg/L for 24 h	Increased mortality, reduced survival, depleted glutathione level, and induced oxidative stress	[75]
	*Ceriodaphnia dubia and Daphnia carinata*	Copper, lead, zinc (at 1.3, 1.1, 13.0 mg/L, respectively) for 48 h or 7 days	Increased mortality and reduced reproduction ability	[123]
Organic compounds	*Daphnia pulex*	Organic selenium at 0.025 mg/L for 48 h	Death, immobility, and molting delay	[122]
	*Daphnia magna*	Propiconazole (pesticide) at 1 μg/L for 4 and 8 days	Impaired adult growth, decreased offspring development, impaired oocyte maturation, and interrupted resulting reproduction	[41]
	*Daphnia magna*	Fenvalerate (insecticide) at 0.6 μg/L for 24 h	Increased mortality, reduced survival, increased arginine phosphate level, and disrupted amino sugar metabolism	[75]
	*Daphnia magna*	Uncoupler of oxidative phosphorylation 2,4-dinitrophenol at 1.5 mg/L for 24 h	Increased mortality, reduced survival, and increased arginine phosphate level	[75]
	*Daphnia magna*	Alkylpolyglucosides (GCP 650, GCP 600, GCP 215) at IC_50_ (29, 14, 111 mg/L, respectively) for 24 h	Increased immobility	[124]
Drugs	*Daphnia magna*	Ibuprofen (non-steroidal anti-inflammatory drug or NSAID) at 20, 40, 80 mg/L for 8 days	Reduced fecundity and arrested early embryogenesis	[104]
	*Daphnia magna*	Beta-blocker anti-hypertension drug propanolol at 1.4 mg/L for 24 h	Increased mortality, reduced survival, and disrupted fatty acid metabolism and eicosanoid biosynthesis	[75]
	*Daphnia magna*	Mefenamicacid at EC_50_ (17.16 mg/L) for 48 h and 1 mg/L for 21 days	Increased immobility and reduced offspring production	[125]
Nanoparticles	*Daphnia magna*	Silver nanoparticles with surface coating at LC_50_ (0.88 μg/L) for 48 h	Increased mortality and reduced survival	[126]
	*Daphnia magna*	Polyvinylpyrrolidone-coated silver nanoparticles at LC_50_ (0.18 mg/L) for 24 h	Increased mortality, reduced survival, disrupted proteolysis and cell cycle	[44]
	*Daphnia magna*	Coated silver nanoparticles (Ag-GAs, Ag-PEGs, and Ag-PVPs) at LC_50_ (3.41, 3.16, 14.81 μg/L, respectively) for 48 h	Increased mortality	[127]
	*Daphnia magna*	Collargol (protein-coated nano Ag) and AgNO_3_ nanoparticles at EC_50_ (20–27 ppb) for 48 h	Increased immobility	[128]
	*Daphnia magna*	Ag and CuO nanoparticles at EC_50_ (3.8 and 2.6 mg/L, respectively) for 24 h	Increased immobility	[129]
	*Daphnia magna*	Titanium dioxide nanoparticles at 0.01–10 mg/L for 48 h	Reduced survival offspring production, and digestion ability	[130]
	*Daphnia magna*	Nano and bulk titanium dioxide at 20 g/L for 48 h	Undetectable toxicity	[131]
	*Daphnia magna*	Bulk CuO, nano CuO and CuSO_4_ at L(E)C_50_ (165, 3.2, 0.17 mg/L, respectively) for 48 h	Increased immobility	[131]
	*Daphnia magna*	Bulk ZnO, nano ZnO and ZnSO_4_·7H_2_O at L(E)C_50_ (1.8, 1.9, 1.1 mg/L, respectively) for 48 h	Increased immobility	[131]

## 4. Perspectives

*Daphnia* offers advantages for toxicological investigation of multiple stressors owing to high growth rate, high sensitivity to environmental changes, wide spatial distribution, parthenogenetic life cycle, and availability of omics-based tools [122]. In particular, its unique parthenogenesis facilitates the study of epigenetic effects without profound genetic differences [132,133]. Epigenetically regulated-sex determination and sexual reproduction are undergone toward harsh environmental conditions.

Mapping the phenotype to the genotype in *Daphnia* system is considerable challenge. Genomic responses to genetic and environmental stressors as well as environmental impact on the phenotype have been pursued [134,135,136]. Integration of genome sequencing with the functional genomic tools provides a better insight on phenotypic evolution, mechanistic elucidation of the evolutionary novel traits, evolutionary adaptation, and regulatory pathways underlying to adaptive evolution.

In the context of ecological risk assessment regarding complex mixtures, genomic tools and fingerprinting approaches offer exceptional platform [40]. Several transcriptomic research projects that use either simple mixtures of particular chemicals or complex mixtures like entire effluents have emerged to evaluate these capabilities, suggesting that the microarray approach was informative [137,138]. A mixture consisting of four chemicals was used to examine the hypothesis that salient transcriptomic responses of individual chemicals could be retained in mixtures, including compounds with dissimilar modes of toxicity [139,140]. This is one of the concepts to applying transcriptome signatures/fingerprints in environmental exposure diagnosis. Metabolomic analyses might complement with phenotypic data or transcriptomic data, while systemic ontologies and pathway analysis software for metabolomics has been developed even less in comparison to transcriptomics and proteomics. However, metabolomics likely has as much potential as transcriptomics or proteomics for utility to ecological risk assessment.

Understanding of genetic and epigenetic mechanisms underlying phenotypic responses to environment (*i.e.*, sex determination, sexual reproduction, helmets, and neckteeth) offers great benefit. In addition to DNA methylation, extensive researches on histone modification, RNAi, and changes in DNA methylation toward environmental stressors throughout developmental stages are necessary. Using *Daphnia* model, the epigenetic discriminatory between sexual and asexual as well as stressor-exposed and non-exposed daphnid individuals will be warranted with potential applications in the area of evolutionary and developmental biology.
